# Determination of Critical Parameters of Drug Substance Influencing Dissolution: A Case Study

**DOI:** 10.1155/2014/929248

**Published:** 2014-09-15

**Authors:** Erika Bojnanska, Michal Kalina, Ladislav Parizek, Eva Bartonickova, Tomas Opravil, Michal Vesely, Miloslav Pekar, Josef Jampilek

**Affiliations:** ^1^Faculty of Pharmacy, University of Veterinary and Pharmaceutical Sciences Brno, Palackeho 1/3, 612 42 Brno, Czech Republic; ^2^Faculty of Chemistry, Brno University of Technology, Purkynova 464/118, 612 00 Brno, Czech Republic

## Abstract

The purpose of this study was to specify critical parameters (physicochemical characteristics) of drug substance that can affect dissolution profile/dissolution rate of the final drug product manufactured by validated procedure from various batches of the same drug substance received from different suppliers. The target was to design a sufficiently robust drug substance specification allowing to obtain a satisfactory drug product. For this reason, five batches of the drug substance and five samples of the final peroral drug products were analysed with the use of solid state analysis methods on the bulk level. Besides polymorphism, particle size distribution, surface area, zeta potential, and water content were identified as important parameters, and the zeta potential and the particle size distribution of the drug substance seem to be critical quality attributes affecting the dissolution rate of the drug substance released from the final peroral drug formulation.

## 1. Introduction

In the past, drug development was primarily empirical and data based [1]. Nowadays pharmaceutical development should be systematic; that is, the process should be understood; a mere change of some parameters of active pharmaceutical ingredients (APIs) of drug formulations so that they comply with some requirements is not sufficient. Process analytical technology (PAT) was defined by the United States Food and Drug Administration (FDA) as a mechanism to design, analyse, and control pharmaceutical manufacturing processes through the measurement of critical process parameters (CPP) which affect critical quality attributes (CQA) and thus by identification of these critical parameters the production process is improved, for example, by in-line or on-line monitoring to minimize production defects [2]. Quality by design (QbD) [1, 3] is connected with PAT. This innovative paradigm is inspired by ICH Q8-Q11 [4–7] and should lead to understanding the effect of incoming material parameters, formulation, and process variables on CQA [8, 9]. Older approved and registered APIs and pharmaceuticals are analysed/controlled by methods used at the time of their registration; that is, their specification cannot include modern analytical methods, such as solid-state analysis. Thus really critical parameters affecting the quality need not be defined in a specification prepared in this manner. This can become a great problem, when API source, site of manufacturing, or manufacturing equipment is changed.

This case study discusses prompt determination of critical parameters of the API purchased from a new supplier to afford the drug formulation to conform to the valid specification. Although the newly supplied API corresponded to the requirements of the current specification, after the change of the API supplier it was found that the drug formulations hardly met the required specification. Therefore the aim of this investigation was to find critical parameters/physicochemical properties of the drug substance that affect the quality of the peroral dosage form. Subsequently these new critical quality attributes were recommended as new parameters for modification of the current API specification. As a result, the composition of the tablet should not be changed, and any change management process should not be initiated. The investigated API contains a tertiary amino group (p*K*_*a*_ ≈ 9) and a carboxyl moiety (p*K*_*a*_ ≈ 4); its lipophilicity expressed as log⁡⁡*P* is ca. 6, and the API can be used as hydrochloride. Due to confidentiality reasons it is not possible to characterise the API more specifically.

## 2. Experimental and Methods

### 2.1. Samples

All the samples of APIs (AS-1–AS-5), that is, five different batches, were received from external suppliers; samples AS-1, AS-2, AS-4, and AS-5 were from a new supplier and sample AS-3 was from a previous supplier. The final drug products, tablet samples TS-1–TS-5, were produced by the same validated manufacturing procedure (preforms with hardness 70–160 N were prepared on a tablet press from the homogenized mixture of the API, diluent, glidant, disintegrant, and calcium stearate; the prepared preforms were milled and then homogenized with calcium stearate, and from the prepared tableting mixture the final tablets were compressed using a tableting machine). It was found that the drug products TS-1 (from AS-1) and TS-2 (from AS-2) provided noncomplying and boundary results of dissolution testing, respectively, while TS-3 (from AS-3), TS-4 (from AS-4), and TS-5 (from AS-5) provided complying results according to the registered drug product specification.

### 2.2. Dissolution Testing

Dissolution tests were performed by a validated method using a USP apparatus 2 (paddle method) Varian Vankel VK 7000 Dissolution System (Agilent Technologies, Santa Clara, CA, USA). Powder samples (180 mg) or one tablet was introduced into 900 mL of dissolution medium (phosphate buffer pH = 7.2) maintained at 37 ± 0.5°C with the rotation speed of 100 rpm. Aliquots of the dissolution samples were withdrawn at predetermined times and filtered through a 0.45 *μ*m PTFE filter. The loss in the volume of the liquid was compensated by addition of fresh dissolution medium maintained at the same temperature. The weight of each drug was used to calculate the dissolved percentage of the drug (Q [%]). The absorbance (drug concentration) of the sample solutions and the reference solution was measured in 1 cm quartz measuring cells at the maximum absorbance wavelength of the drug against the dissolution medium using a Varian Cary 50 UV-VIS Spectrophotometer (Agilent Technologies, Santa Clara, CA, USA). The measurements were repeated six times. All the presented results are reported as the mean value of these six independent measurements (mean ± SD, *n* = 6). All the results are summarized in Table 1.

### 2.3. Water Content

The determination was performed by a titrator 701 KF Titrino (Methrom, Herisau, Switzerland) according to the Ph.Eur 7.7, Chapter 2.5.32 (Water: Micro determination) using 200 mg of the API dissolved in 1 mL of dry methanol. The measurements were repeated three times. All the presented results are reported as the mean value of these three independent measurements (mean ± SD, *n* = 3). All the results are summarized in Table 2.

### 2.4. Wettability—Water Contact Angle

The tablets were made from each powder sample in a standard press for infrared spectroscopy tablets by the power of 80 kN for 20 seconds. Droplets (5 *μ*L) of phosphate buffer (pH = 7.2) were used as the medium. The water contact angles were measured at 25°C by OCA20 (DataPhysics, Applied Photophysics, UK) using the sessile drop method. Sixteen values of the contact angle were measured and statistically evaluated. All the results are summarized in Table 2.

### 2.5. Zeta Potential

The zeta potential of the samples was obtained using Zetasizer Nano ZS (Malvern Instruments, UK) by means of electrophoretic light scattering. The zeta potential of the samples was measured in glass cuvettes with an inserted dip cell. The instrument detects the Doppler shift between the laser beam (5 mW, 633 nm) passing through the cuvette with the sample and the reference beam passing outside the cuvette. The data are evaluated using phase analysis light scattering. The samples were prepared by dissolving solid powder in phosphate buffer (pH = 7.2) to reach their final concentration 0.2 mg/mL. The measuring was carried out at laboratory temperature (25.0 ± 0.1°C). The measurements were repeated four times. All the presented results of the zeta potential of the samples are reported as the mean value of these four independent measurements (mean ± SD, *n* = 4). All the results are summarized in Table 2.

### 2.6. Particle Size Measurement

The particle/agglomerate size of samples was measured by means of a laser diffraction analyser HELOS/KR (SympaTec, Clausthal-Zellerfeld, Germany) using the combination of MIEE and Fraunhofer theory for calculation. The measured data were statistically calculated from the measurements by 3 types of objectives (range from 100 nm to 30 mm). The measurements were repeated five times. All the presented results of the particle size samples are reported as the mean value of these five independent measurements (mean ± SD, *n* = 5). All the results are summarized in Table 2.

### 2.7. Specific Surface Area

The values of specific surface area (SSA) were determined using a Quantachrome NOVA 2200 analyser (Quantachrome Instruments, Boynton Beach, FL, USA). The samples were degassed using vacuum under the temperature of 90°C for 3 h. The degassed samples were placed into the measuring chamber and evacuated under pressure around 1.33 × 10^−6^ MPa. The evacuated chamber was filled with pure nitrogen with partial pressure* p*/*p*_o_ varied from 0.03 to 0.5. The obtained data (from full absorption and desorption) were fitted by using Brunauer-Emmet-Teller isotherm [10] to determine specific surface values. The measurements were repeated three times. All the presented results of the SSA of the samples are reported as the mean value of these three independent measurements (mean ± SD, *n* = 3). All the results are summarized in Table 2.

### 2.8. Scanning Electron Microscopy

The morphologies of the samples were examined using a scanning electron microscope (SEM). The Au sputtering was applied to avoid unfavourable sample conductivity. The samples were analysed using the scanning electron microscope equipped with an EDX analyzer (Zeiss EVO LS10, Zeiss, Germany) and using accelerating voltage 10.0 kV, working distance 8.0 mm, and probe current 100 pA.

### 2.9. Statistical Analysis

All experiments were repeated and the data were expressed as means ± SD. The differences were evaluated by one-way analysis of the variance (ANOVA) completed with the Bonferroni's multicomparison test (ORIGIN PRO7). The differences (marked by different small letters) were considered significant at *P* = 0.05.

## 3. Results and Discussion

**Figure 1:**
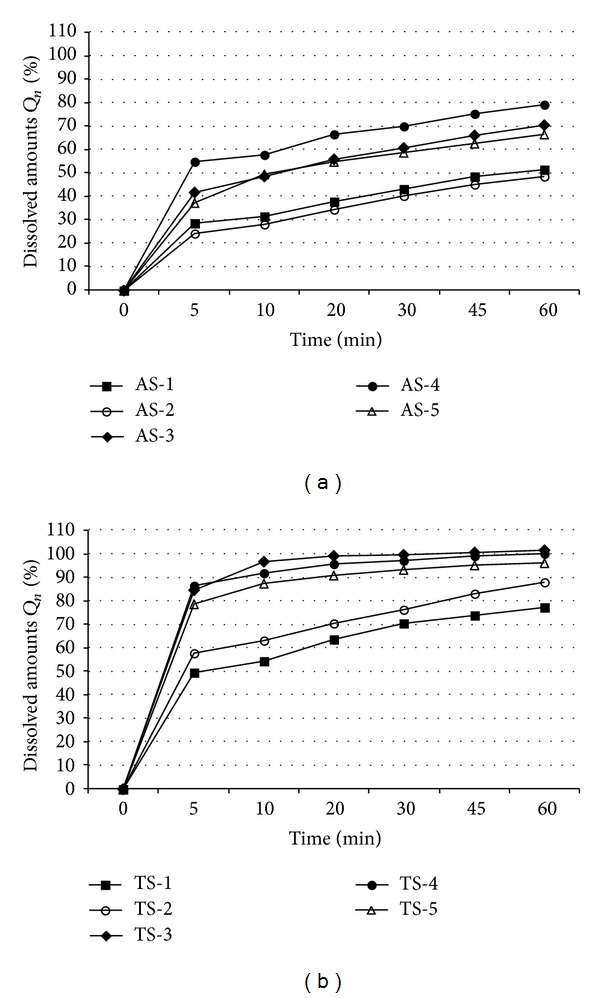
Dissolution profiles (dissolved amounts *Q*_*n*_ [%]) of individual API samples AS-1–AS-5 (a) and individual tablets TS-1–TS-5 (b). *Q*_*n*_ values are expressed as mean ± SD (*n* = 6 units). SDs are not illustrated to improve visualisation.

A new supplier provided two batches of API (AS-1 and AS-2) that gave noncomplying results of dissolution testing of the drug product, tablet sample, TS-1, and boundary results of dissolution testing of tablet sample TS-2. The results of these analyses were verified by comparison with the results of dissolution of tablet sample TS-3 that was manufactured under the same validated procedure as samples TS-1 and TS-2 from API sample AS-3 purchased from the previous supplier. Tablet sample TS-3 met all the criteria of the valid drug product specification. Based on these observation patterns, API batches AS-1, AS-2, and AS-3 were also tested for their dissolution profiles/dissolution rate and great differences were found; see Figure 1. The subsequent investigation of samples AS-1–AS-3 was started by determination of polymorphism by powder X-ray diffraction (PXRD). It can be stated that all evaluated API samples contained the same polymorph (diffractograms are not presented due to confidentiality reasons). Although both new samples AS-1 and AS-2 met the criteria of the API specification for the first time, the change of the quality of new API samples was found in the production of the drug formulation; the new samples showed considerable adhesiveness and electrostatic charge in comparison with the API from the previous supplier. In the light of the below discussed results it can be supposed that these properties are connected with particle size distribution. Additionally other parameters such as zeta potential, particle size, surface area, water content, and water contact angle of all three batches of the API (AS-1, AS-2, and AS-3) were tested. Based on the obtained preliminary results some recommendations were made for modification of API parameters. Subsequently two new batches of the API from the new supplier manufactured according to these criteria and the tablets produced from these APIs (AS-4/TS-4 and AS-5/TS-5) were subjected to all the above mentioned tests, and critical parameters were suggested. Although the total number of samples was limited, some noteworthy relationships between the properties/parameters of APIs and their dissolution were found.

The comparison of dissolution profiles (dissolution amounts *Q*_*n*_ [%]) of individual samples is shown in Table 1 and illustrated in Figure 1. The dissolution testing of the API batches (AS-1–AS-5) and the tablets (TS-1–TS-5) was performed according to the valid specification in phosphate buffer (pH = 7.2). The same medium and concentration were used for determination of zeta potential and wetting (contact) angle; see below. It is evident that *Q*_*n*_ values of the pure API are significantly lower (see Figure 1(a)) than *Q*_*n*_ values of APIs from the tablets (see Figure 1(b)). This fact can be caused by the excipient (calcium stearate) that facilitates solubilisation of the API. Different behaviour of AS-1 and AS-2 in comparison with AS-3, AS-4, and AS-5 was observed. Both AS-1 and AS-2 stayed on the surface nonwetted, while the others sank and dissolved. From the data presented in Figures 1(a) and 1(b) it can be assumed that if the amount of the dissolved API achieved in the 10th minute (*Q*_10_) approximately equals to 50%, the dissolution testing of the tablets will comply with the valid specification (*Q* = 75% of the stated amount after 45 min).

All the experimental results were compared by means of statistical analysis. The results of one-way analysis of the variance (ANOVA) completed with the results of the Bonferroni's multicomparison test are presented in Table 1, where differences were considered significant at *P* = 0.05. Significant differences between the *Q*_*n*_ values determined for samples AS-1/TS-1 and AS-2/TS-2 and complying samples AS-3/TS-3–AS-5/TS-5 were estimated.

**Figure 2:**
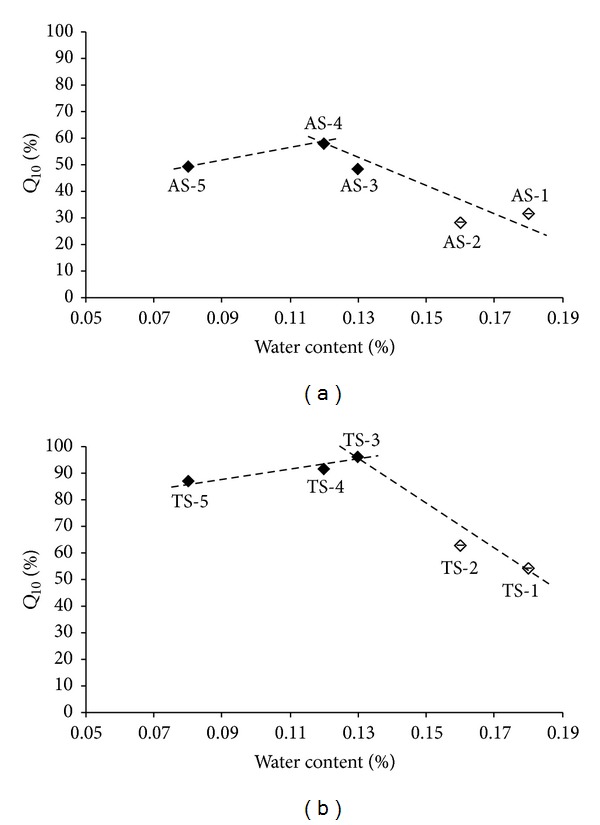
Dependence of dissolved amounts *Q*_10_ [%] of individual API samples AS-1–AS-5 (a) and individual tablets TS-1–TS-5 (b) in the 10th minute on water content [%]. Samples with boundary values AS-1, AS-2, TS-1, and TS-2 are marked by empty symbols. The data represent the mean ± SD of three samples.

**Figure 3:**
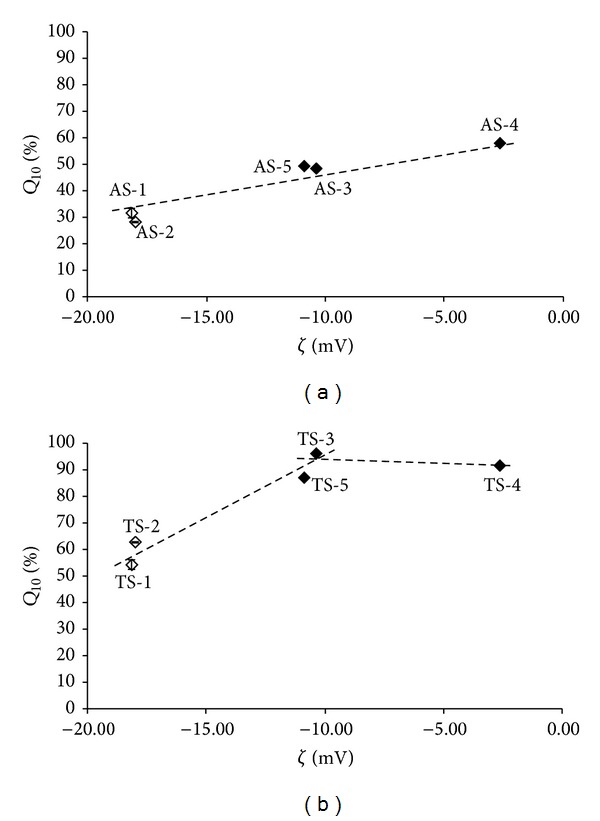
Dependence of dissolved amounts *Q*_10_ [%] of individual API samples AS-1–AS-5 (a) and individual tablets TS-1–TS-5 (b) in the 10th minute on zeta potential values (*ζ* [mV]). Samples with boundary values AS-1, AS-2, TS-1, and TS-2 are marked by empty symbols. The data represent the mean ± SD of four samples.

**Figure 4:**
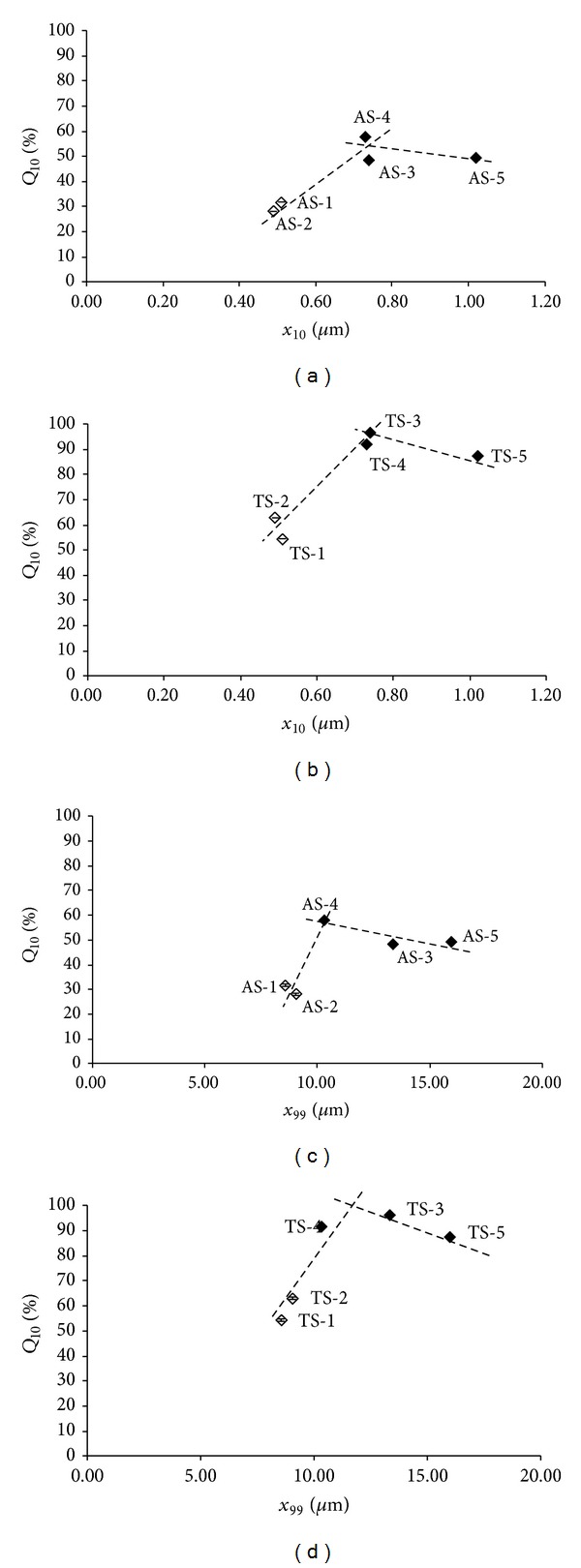
Dependence of dissolved amounts *Q*_10_ [%] of individual API samples AS-1–AS-5 ((a), (c)) and individual tablets TS-1–TS-5 ((b), (d)) in the 10th minute on particle size (*x*_10_ and* x*_99_ [*μ*m]). Samples with boundary values AS-1, AS-2, TS-1, and TS-2 are marked by empty symbols. The data represent the mean ± SD of five samples.

From the slope of the curves of the graphs in Figures 1(a) and 1(b), that is, from the dissolution rate, it seems that the pure or formulated API is most rapidly dissolved by the 5th minute. Within subsequent 5 minutes the rate of dissolution decreased; nevertheless, it can be stated that in comparison with the following time interval (from the 10th to the 60th minute) the amount of the API dissolved within the first 10 minutes is the most important, especially for the formulation and for satisfactory results of dissolution testing. For this reason all the graphs presented below (Figures 2–4 and 6) illustrate the amount of the dissolved API in the 10th minute (*Q*_10_). It is important to note that the dependence shown and discussed below is obviously valid also for dissolved amount *Q*_*n*_ in other measured time-intervals.

**Figure 5:**
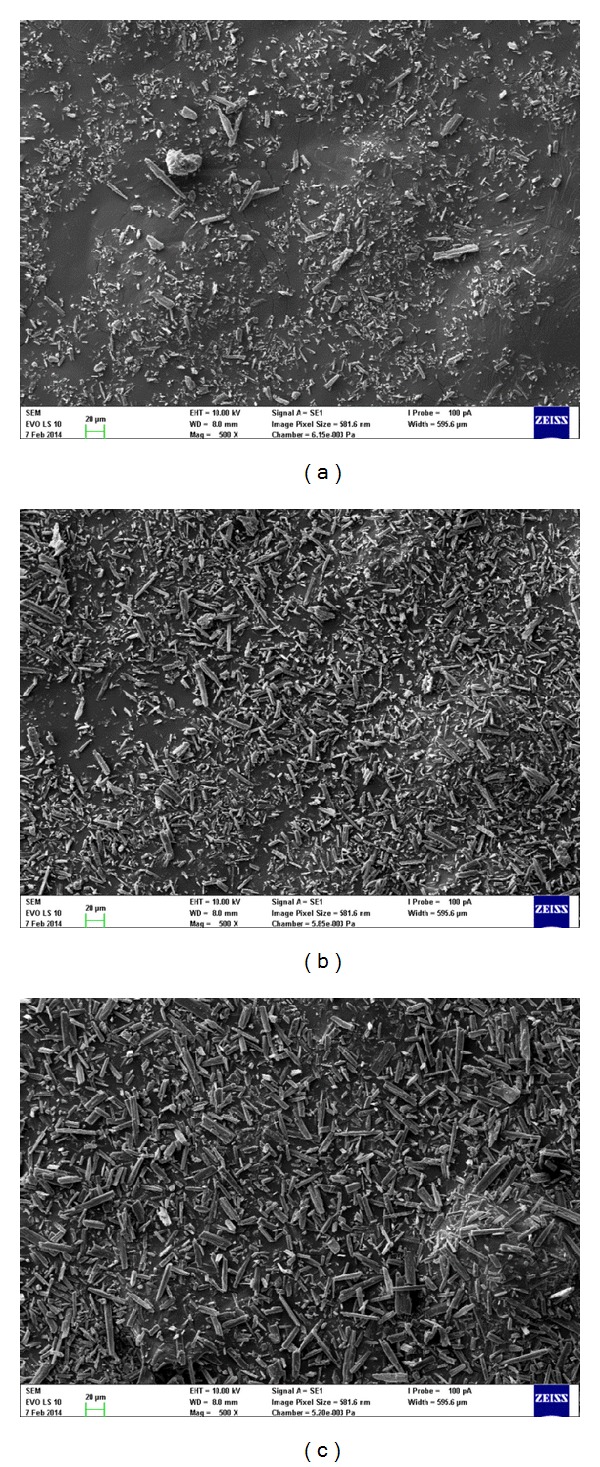
SEM microphotographs of noncomplying sample AS-1 (a) and complying samples AS-3 (b) and AS-5 (c).

**Figure 6:**
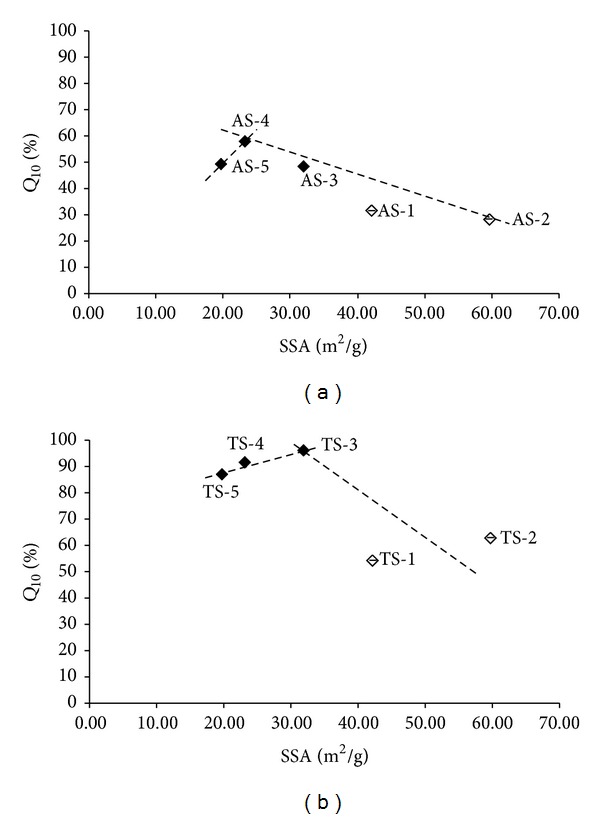
Dependence of dissolved amounts *Q*_10_ [%] of individual API samples AS-1–AS-5 (a) and individual tablets TS-1–TS-5 (b) in the 10th minute on SSA [m^2^/g]. Samples with boundary values AS-1, AS-2, TS-1, and TS-2 are marked by empty symbols. The data represent the mean ± SD of three samples.

As mentioned above, API samples AS-1–AS-5 were evaluated for their water content, wettability, zeta potential, particle size, and specific surface area. Also the shapes of individual API samples were investigated by scanning electron microscope analysis. All these results are presented in Table 2 and illustrated in Figures 2–6.

The results of one-way analysis of the variance (ANOVA) completed with the results of the Bonferroni's multicomparison test are presented in Table 2, where differences were considered significant at *P* = 0.05. Significant differences between water content, zeta potential, particle sizes, and surface area for samples AS-1/TS-1 and AS-2/TS-2 and complying samples AS-3/TS-3–AS-5/TS-5 were determined. No statistical significance was found for samples AS-1, AS-2, AS-4, and AS-5 with regard to water contact angle.

The bilinear dependence of the *Q*_10_ values on water content in APIs is shown in Figure 2. It is evident that the *Q*_10_ values of APIs AS-1 and AS-2 that afforded noncomplying formulations TS-1 and TS-2 are low and represent remote points. The decreasing dependence in Figure 2(a) has correlation coefficient = −0.9140(*n* = 4); the increasing dependence in Figure 2(b) has correlation coefficient *r* = 0.9406(*n* = 3), and the decreasing dependence in Figure 2(b) has *r* = −0.9772(*n* = 3). The optimum seems to be 0.12-0.13% of water. It can be stated that the samples with water content higher than 0.13% can stop complying in relation to dissolution testing. Unambiguously it can be found that the API with water content higher than 0.16% does not comply.

Wettability expressed as water contact angle (*θ* [°]) unfortunately did not provide the required information. From the results presented in Table 2 it is apparent that all the samples of APIs are wetted (*θ* < 90° involves compounds with good wettability) [11]. All angles were up to 35°; AS-3 is a drug substance with excellent wettability that caused the API to absorb water too fast.

Generally, zeta potential is used for determination of stability of colloidal systems. A high zeta potential (*ζ* > ±30 mV) is an indicative of the system stability (resistance to aggregation). When the potential is small (interval from 0 to ±30 mV), attractive forces may exceed this repulsion and the system tends to coagulate [12]. Nevertheless, as phosphate buffer with pH = 7.2 and the concentration of the API were chosen for the measurement of zeta potential to reflect real conditions at dissolution testing, and as the investigated compounds can be considered as zwitterions (tertiary amino and carboxyl moieties), it can be supposed that the value of pH = 7.2 is close to the point of zero charge. At this point, when the compounds have zero zeta potential, minimum stability, maximum solubility of the solid phase, and other peculiarities are demonstrated [13, 14]. The zeta potential values of boundary samples AS-1 and AS-2 (*ζ* ≈ −18 mV) are significantly different from those of complying samples AS-3, AS-4, and AS-5 (*ζ* ≈ −3 mV, *ζ* ≈ −11 mV); see Table 2, and it can be stated that the samples with zeta potential close to zero expressed significantly higher solubility. The dependence of the *Q*_10_ values of the samples on zeta potential is illustrated in Figure 3. The dependence of the dissolution of the pure API on the zeta potential seems to be linear-increasing (correlation coefficient *r* = 0.9654, *n* = 5) with the zeta potential close to 0 mV (see Figure 3(a)), while for the dissolution of the API from the tablets, a biphasic course was found (Figure 3(b)). Up to an optimum *ζ* ≈ −11 mV a linear increase of *Q*_10_ was observed (*r* = 0.9774, *n* = 4), and further increase in zeta potential did not affect significantly the dissolved amount. The different course of the dependence illustrated in Figures 3(a) and 3(b) can be explained by the influence of excipients on the API. Based on the data it can be concluded that the more different the values of zeta potential are from zero, the less soluble the substances are. Despite the limited number of the samples, the values of zeta potential ranged from 0 to −11 mV can be considered as advisable for good solubility.

Figure 4 shows the dependence of the *Q*_10_ values of the individual API samples (Figures 4(a) and 4(c)) or the dissolved API released from individual tablets (Figures 4(b) and 4(d)) on particle size. The terminal particle sizes* x*_10_ and* x*_99_ were chosen for highlighting this dependence. Bilinear curves can be observed for the dependence of dissolution on particle size with an optimum of particle size* x*_10_ ≈ 0.74 *μ*m and* x*_99_ ≈ 10–13 *μ*m. It is possible to see that the total increase of particle size distribution profile of the substance causes less significant effect on the amount of the dissolved compound than a particle size reduction, especially in Figures 4(b) and 4(d) illustrating the dissolution of the API from the tablets (compare samples 1, 2, and 5). Although reduced particle size aids the formulation of poorly water soluble APIs [15], in this case, a large share of small particles results in a significant decrease of dissolution and probably also causes processability difficulties as mentioned above. The significant decrease of the dissolved API with a large share of small particles can be also related to particle surface area; see below. Based on the results listed in Table 2 and illustrated in Figure 4, it can be stated that particle size limits should be 0.8–1.0 *μ*m for* x*_10_, 2.4–5.1 *μ*m for* x*_50_, and 11–16 *μ*m for* x*_99_.

The investigation of particle size distribution is closely connected with shape analysis of the particles. The SEM analysis of all the API samples supported the results of particle size distribution analysis. The microphotographs of samples AS-1, AS-3, and AS-5 with 500x magnification are presented in Figure 5. The general shape of all particles can be considered as tabular or laths. The laths of AS-1 and AS-2 are very fine, those of AS-4 are medium, and those of AS-3 and AS-5 are coarse.

The specific surface area (SSA) is often correlated with the rates of dissolution. The SSA is increased with decreasing particle size and with increasing porosity of the particles. The generation of porosity, especially in the case of small pores, can produce SSA far in excess of that produced by particle size reduction. The SSA influences processing and behaviour of powders and porous solids, since the surface area corresponds to the roughness of the particle exterior and its porous interior [16, 17]. One of the possibilities to measure SSA is using the BET approach [10]. The dependence of the *Q*_10_ values of the individual samples of the API or the API released from the tablets on the SSA (porosity) expressed as SSA values [m^2^/g] is illustrated in Figure 6. Both boundary APIs 1 and 2 (samples AS-1/TS-1 and AS-2/TS-2) represent the remote points in Figures 6(a) and 6(b), where bilinear courses can be found again; the correlation coefficient of the decreasing part of the course in Figure 6(a) is *r* = −0.9299(*n* = 4) and the increasing part of the course in Figure 6(b) is = 0.9742(*n* = 3). According to the results SSA ≈ 23 m^2^/g (AS-4) is preferable for the dissolution of pure API, while SSA ≈ 32 m^2^/g (AS-3) is favoured for the dissolution of the drug formulation. As mentioned above, a slight change of the dissolution rate/amount of the pure API in comparison with the formulated API is caused by an excipient. Apparently for both cases it can be summarized that for satisfactory dissolution testing the specific surface area should be in the range 20–32 m^2^/g. An increase of the surface area (porosity) leads to a rapid decrease of dissolution.

Similar results can be found for some drugs, particularly for those that are lipophilic in nature, where particle size reduction can result in aggregation of the material. This causes a consequent reduction in the effective surface area of the drugs exposed to liquids and hence a reduction in their dissolution rate. For example, acetylsalicylic acid, phenacetin, or phenobarbital is prone to aggregation in particle size reduction. Thus, although a particle size reduction and an increase of the surface area are recommended to increase the dissolution rate of drugs in general, for some APIs, the improvement of solubility by particle size reduction/increase of the surface area ceases when the particle size reaches a particular value. Hence, the particle size is critical and beyond a particular value the solubility of solid substances decreases. It can be stated that such changes can arise because of the presence of an electrical charge on the particle, which is predominant in small particles [17–20]. Based on the above-mentioned facts zeta potential and particle size were evaluated as critical for satisfactory dissolution.

## 4. Conclusions

A change of the supplier of the API for an older medicament led to noncomplying results of the dissolution test (samples TS-1 and TS-2). Firstly a sample of the API from the original supplier (AP-3) and two different samples of the API from the new supplier (AS-1 and AS-2) were analysed in detail. The sample of the API from the original supplier and its final peroral dosage form were used as standards (AP-3 and TS-3), and based on the parameters of this API (sample AS-3) some criteria were advised. The results of analytical evaluation of other two samples/batches of the APIs (AS-4 and AS-5) and the tablets manufactured therefrom (samples TS-4 and TS-5) confirmed the presumptions and led to the following recommendations. Based on the performed analytical testing, finding and interpretation of relationships between the amount of soluble pure API/API released from the tablets and the determined physicochemical parameters, particle size, surface area, zeta potential and, perhaps, water content can be considered as critical parameters affecting the dissolution rate of the final product. It can be summarized that for satisfactory dissolution test of the final product the specification of the API should be modified and completed as follows. Zeta potential should be in the range from 0 to −11 mV, particle size limits should be* x*_10_ > 0.80 *μ*m and* x*_99_ < 13 *μ*m, specific surface area (BET) should be in the range 19–32 m^2^/g, and the water content in the API should not be higher than 0.13%, provided that similar instrumentation and the same experimental conditions of measurement are chosen.

## Figures and Tables

**Table 1:** Dissolved amounts *Q*_*n*_ [%] of pure API and API from tablets. *Q*_*n*_ values are expressed as mean ± SD (*n* = 6 units). The means followed by different letters are significantly different at *P* = 0.05.

Sample	Dissolved amounts *Q*_*n*_ [%]					
	5 min	10 min	20 min	30 min	45 min	60 min
AS-1	28.5 ± 3.2^a^	31.6 ± 4.0^a^	37.8 ± 4.0^a^	43.0 ± 4.3^a^	48.2 ± 3.9^a^	51.4 ± 4.0^a^
AS-2	24.3 ± 3.9^a^	28.2 ± 4.0^a^	34.5 ± 4.8^a^	40.1 ± 5.0^a^	45.2 ± 6.1^a^	48.4 ± 5.7^a^
AS-3	41.7 ± 0.3^c^	48.5 ± 0.4^b^	55.8 ± 0.5^b^	60.7 ± 0.5^b^	65.7 ± 0.3^c^	70.1 ± 0.4^c^
AS-4	54.8 ± 0.4^e^	57.9 ± 2.5^c^	66.5 ± 2.7^cd^	69.8 ± 1.5^c^	75.2 ± 0.9^d^	79.3 ± 1.3^d^
AS-5	37.3 ± 0.3^b^	49.3 ± 0.5^b^	54.6 ± 2.3^b^	58.5 ± 1.6^b^	62.4 ± 1.1^b^	66.6 ± 1.1^b^
TS-1	49.3 ± 3.2^d^	54.3 ± 2.1^c^	63.4 ± 2.2^c^	70.4 ± 2.3^c^	73.7 ± 2.7^d^	76.9 ± 2.0^d^
TS-2	57.5 ± 1.2^f^	62.9 ± 1.1^d^	70.4 ± 1.2^cd^	76.2 ± 1.4^d^	83.0 ± 1.5^e^	87.8 ± 1.9^e^
TS-3	84.6 ± 1.9^h^	96.4 ± 2.1^e^	98.8 ± 2.2^g^	99.7 ± 2.1^f^	100.7 ± 2.4^g^	101.4 ± 2.1^g^
TS-4	86.2 ± 1.2^h^	91.8 ± 0.9^f^	95.4 ± 0.8^f^	97.2 ± 0.8^f^	98.8 ± 0.9^g^	100.0 ± 0.8^g^
TS-5	78.4 ± 1.7^g^	87.4 ± 2.4^e^	90.7 ± 2.1^e^	93.1 ± 1.8^e^	95.1 ± 1.8^f^	96.3 ± 1.6^f^

**Table 2:** Other determined characteristics of investigated samples of APIs AS-1–AS-5: water content [%], wettability (water contact angle *θ* [°]), zeta potential (*ζ* [mV]), particle size (*x*_10_, *x*_50_, *x*_90_ [*µ*m]), and specific surface area (SSA [m²/g]). Values are expressed as mean ± SD (see Section 2 for number of experiments for individual methodology). The means followed by different letters are significantly different at *P* = 0.05.

Sample	Water [%]	*θ* [°]	*ζ* [mV]	Particle size [*µ*m]			SSA [m²/g]
				*x*_10_	*x*_50_	*x*_99_	
AS-1	0.18 ± 0.01^c^	35.6 ± 1.7^b^	−18.13 ± 1.94^a^	0.51 ± 0.01^a^	2.12 ± 0.01^a^	8.57 ± 0.37^a^	42.11 ± 0.03^d^
AS-2	0.16 ± 0.01^c^	34.9 ± 3.1^b^	−17.98 ± 0.34^a^	0.49 ± 0.01^a^	2.18 ± 0.12^b^	9.06 ± 0.39^b^	59.65 ± 0.04^e^
AS-3	0.13 ± 0.01^b^	11.0 ± 6.6^a^	−10.36 ± 0.40^b^	0.74 ± 0.01^b^	3.15 ± 0.08^d^	13.36 ± 0.19^d^	31.91 ± 0.04^c^
AS-4	0.12 ± 0.01^b^	35.2 ± 0.7^b^	−2.64 ± 0.17^c^	0.73 ± 0.06^b^	2.36 ± 0.04^c^	10.33 ± 0.14^c^	23.21 ± 0.05^b^
AS-5	0.08 ± 0.01^a^	33.4 ± 2.7^b^	−10.90 ± 0.37^b^	1.02 ± 0.03^c^	5.05 ± 0.05^e^	15.97 ± 0.24^e^	19.69 ± 0.06^a^
